# CT and MRI Findings of Soft Tissue Adult Fibrosarcoma in Extremities

**DOI:** 10.1155/2018/6075705

**Published:** 2018-03-06

**Authors:** Hexiang Wang, Pei Nie, Cheng Dong, Jie Li, Yonghua Huang, Dapeng Hao, Wenjian Xu

**Affiliations:** ^1^Department of Radiology, The Affiliated Hospital of Qingdao University, Qingdao, Shandong, China; ^2^Department of Radiology, Puyang City Oilfield General Hospital, Puyang, Henan, China

## Abstract

**Objective:**

To characterize and evaluate CT and MRI features of extremity soft tissue adult fibrosarcoma.

**Methods:**

CT and MRI images from 10 adult patients with pathologically proven fibrosarcomas were retrospectively analyzed with regard to tumor location, size, number, shape, margins, attenuation, signal intensity, and enhancement patterns on MR images. Additionally, the relationships between lesions, deep fascia, and change in adjacent bones were also assessed.

**Results:**

Nineteen tumor lesions in 10 patients were selected for this study. Eighteen lesions were lobulated and one was oval in shape. Most cases were located under the deep fascia, including seven cases that had a nodular lump adjacent to the deep fascia and one case that had broken lesion through the deep fascia. On CT, the adult fibrosarcomas mostly showed iso-attenuated soft tissue masses (*n* = 6). On MRI, all the cases (*n* = 9) displayed low signal on T1-weighted imaging (T1WI) and heterogeneous low and high intensity signals on T2-weighted imaging (T2WI), with band-like areas of low signal on both T1WI and T2WI. On contrast-enhanced MRI images, three cases showed heterogeneous peripheral enhancement and one case demonstrated a spoke-wheel-like enhancement. Eight cases showed muscle edema signals in the peritumoral muscle and one case involved adjacent bone.

**Conclusion:**

CT and MR imaging have respective advantages in diagnosing adult fibrosarcoma. Combined application of CT and MR is recommended for patients with suspected adult fibrosarcoma.

## 1. Introduction

Adult fibrosarcoma is a rare neoplasm composed of malignant spindle-shaped fibroblasts with variable collagen production. In classic cases, these neoplasms are arranged in herringbone patterns and were once considered to be the most common soft tissue sarcoma in adults [1]. However, over the past seven decades the incidence of fibrosarcoma has declined dramatically; according to a recent survey, epidemiological and end results data showed that fibrosarcomas only accounted for 1–3.6% of soft tissue sarcomas in adults [2, 3]. By age and prognosis, fibrosarcomas are divided into adult and infantile fibrosarcomas. Infantile fibrosarcomas are generally considered to be defined as those that occur before the age of 15 [4]. However, some scholars only consider cases diagnosed before the age of two to be infantile fibrosarcomas [5]. Adult fibrosarcomas most commonly occur in the deep soft tissues of the trunk and the upper and lower extremities [6]. In this study, we retrospectively analyzed CT and MRI results of a group of 10 patients with extremity soft tissue adult fibrosarcoma, to gain a deeper understanding of the condition. To the best of our knowledge, this work is the first systematic study of extremity soft tissue adult fibrosarcoma.

## 2. Materials and Methods

### 2.1. Patients

This study, which was approved by our institutional ethics committee, retrospectively analyzed CT (6/10) and MRI (9/10) scans obtained from ten patients (two males and eight females) with pathologically proven adult fibrosarcoma who were imaged between July 2004 and January 2016. The patients had an average age of 50.2 years (range: 18–75 years). The course of disease in the sample pool varied from three months to six years. All patients had painless soft tissue masses as the first symptom. Nine cases were followed up for periods of 2–37 months. Of these, six cases had a recurrence and three cases a lung metastasis. All ten patients underwent surgery. Six patients were referred for CT scanning and nine for MR imaging.

The CT scan parameters were as follows: 2–5 mm axial CT scanning using a soft tissue algorithm, 25–40 cm field of view, 120 kV voltage, 250–300 mA current, and 512 × 512 matrix. All images were reformatted using a soft tissue algorithm and/or bone algorithm. Contrast media were not administered. Reformatted coronal, sagittal, and volume-rendered images were also obtained (*n* = 4).

MRI examinations were performed using either 1.5-T (*n* = 4; Signa Advantage Horizon; GE Medical Systems, Milwaukee, WI, USA) or 3.0-T MRI scanner (*n* = 5; Signa HDx; GE Medical Systems). T1-weighted (T1W) SE (TR 500–600 ms, TE 10–15 ms) and fat suppressed T2-weighted (T2W) FSE (FS-FSE T2WI, TR 3600–4500 ms, TE 80–120 ms) sequences were used, with a slice thickness of 4 mm and a slice spacing of 0.5–1 mm. Two patients underwent diffusion-weighted imaging (*b* = 0 and 800 s/mm^2^) and four underwent a contrast-enhanced MR scan using a T1W SE sequence (TR 500–600 ms, TE 10–15 ms) with an injection of 0.2 ml/kg gadolinium dimeglumine.

### 2.2. Imaging Analysis

Two musculoskeletal radiologists with more than ten years of professional experience and blinded to the clinical and pathological findings independently reviewed the imaging. The readers recorded the tumor location (in the deep muscle group or under the deep fascia), size (maximum diameter of tumor), morphology (oval, lobulated, or irregular), number of lesions (single or multiple), edges (clear or unclear boundaries), attenuation, signal intensity, the presence of cystic and/or necrotic tissue, and the presence of a fibrous septum (band-like areas of low signal on both T1WI and T2WI, without enhancement on MRI, and a soft tissue density similar to muscle on plain CT). On the plain CT and MR images, attenuation or intensity was classified as hypo, iso, or hyper in comparison with adjacent muscle. When contrast was administered on MRI, the degree of enhancement was classified as no enhancement, mild, moderate, or marked. Other associated signs, including the relationship between the lesions and the deep fascia (whether adjacent to the deep fascia, with or without a nodular lump, and whether it broke through the deep fascia) and whether the adjacent structures (bone, muscle, nerves, or blood vessels) had been invaded, were also recorded. The independent imaging findings were compared with the pathologic findings.

## 3. Results

**Figure 1:**
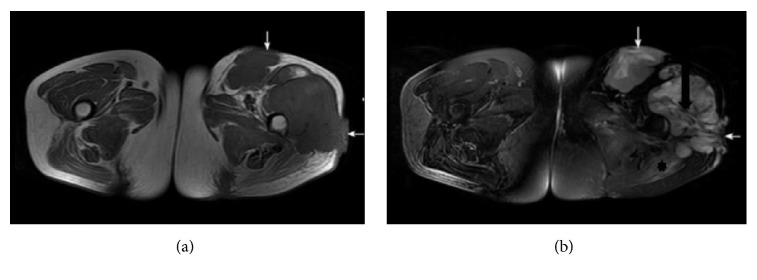
*A 43-year-old female with a moderately differentiated adult fibrosarcoma*. (a, b) Axial SE T1WI and fat suppressed FSE T2WI, exhibiting two lobulated long T1 mixed T2 signal masses in the left hip deep fascia. The band-like areas of low signal suggest tumor fibrous septa (black arrow in (b)), and the patch-shaped regions of long T1 and T2 signal in the left gluteus maximus muscle represent muscle edema (asterisk in (b)). The lesion broke through the deep fascia (white arrows in (a), (b)).

**Figure 2:**
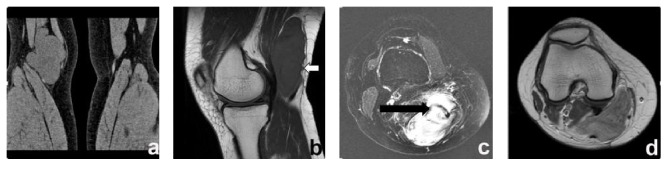
*A 47-year-old female with well-differentiated adult fibrosarcoma*. (a) A coronal CT scan exhibited a lobulated iso-attenuating soft tissue mass in the right popliteal fossa. (b) Sagittal T1WI showed the lesion with a nodular lump close to the deep fascia (white arrow). (c) Axial fat suppressed FSE T2WI showed band-like areas of low signal suggesting tumor fibrous septa (black arrow). (d) An axial SE T1W contrast-enhanced scan exhibited heterogeneous peripheral enhancement.

**Figure 3:**
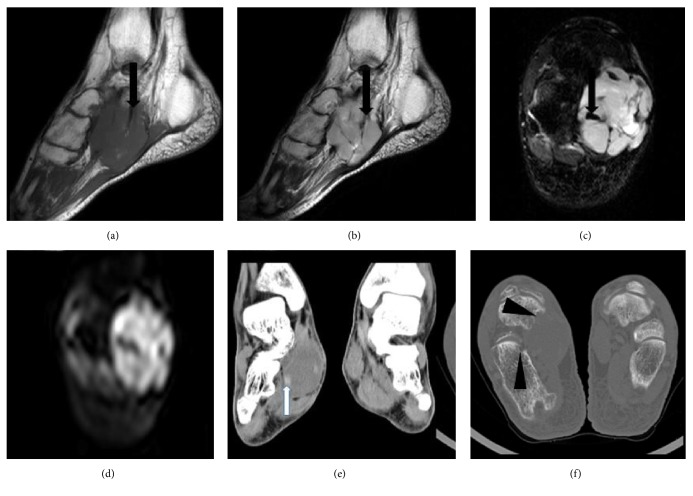
*A 46-year-old female with a poorly differentiated adult fibrosarcoma*. (a, b) Sagittal T1WI and T2WI exhibited a lobulated long T1 and mixed T2 signal mass in the right plantar deep fascia, with band-like areas of long T1 and short T2 signal (black arrows in (a) and (b)) indicating tumor fibrous septa. (c, d) Axial fat suppressed FSE T2WI and DWI show mixed signals, with more nodular short T2 signals (black arrow in (c)). The high lesion signal on DWI indicated dense cellularity. (e, f) Reformatted coronal images of soft tissue and axial bone images displaying an iso-attenuating soft tissue mass, with a slightly higher density small nodule (white arrow in (e)) and partial bone destruction (arrow heads in (f)).

**Figure 4:**
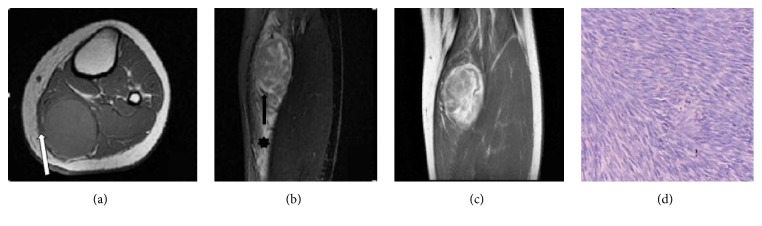
*An 18-year-old female with a moderately differentiated adult fibrosarcoma*. (a, b) Axial SE T1WI and coronal fat suppressed FSE T2WI exhibiting lobulated long T1 and mixed T2 signal masses in the left calf deep fascia, with a nodular lump close to the deep fascia (white arrow in (a)). T2WI exhibited band-like areas of low signal (black arrow in (b)) with patch-shaped long T2 signal regions in surrounding muscle, representing muscle edema (asterisk in (b)). (c) Coronal SE T1W enhanced scan showing a spoke-wheel-like enhancement pattern. (d) Pathological image (He ×100), with tumor cells and collagen fibers arranged in crisscrossing bundles, demonstrating a characteristic “herringbone” arrangement.

The clinical findings in the ten patients with adult fibrosarcoma are summarized in Table 1. This study investigated ten patients with a total of 19 lesions, with eight patients having a solitary lesion and two having multiple lesions (one case had two lesions and the other had nine lesions). Five cases were located in the hip area, two in the legs, one in the popliteal fossa, one in the foot, and one in the arm. Eighteen lesions in nine different cases were lobulated, with the other lesion being oval. In eight cases, the long axes of the lesions were parallel with the long axis of the patient's body. The diameter of the largest lesion was 10.5 cm, the smallest was 1.2 cm, and the average value was 6.7 cm. Eight cases were located in the deep fascia (Figures 1–4) and two in muscle gaps. The margins of all lesions were clear.

The CT and MRI features of the 10 patients with adult fibrosarcoma are summarized in Table 2. Six of the ten patients were examined using CT. On CT with a soft tissue window, all of the lesions exhibited iso-attenuating soft tissue masses (Figures 2(a) and 3(e)), one with patches of slight hypoattenuation and another with some nodes of slight hyperattenuation (Figure 3(e)).

Nine patients underwent MRI examinations, with T1WI demonstrating a low signal (Figures 1(a), 2(b), 3(a), and 4(a)) and T2WI exhibiting mixed signals (Figures 1(b), 2(c), 3(b), 3(c), and 4(b)). There were band-like areas of low signal on both nonenhanced T1WI and T2WI, but these were more substantial on T2WI (Figures 1–4). Eight cases demonstrated patch-shaped regions of long T1 and T2 signal in the peritumoral muscle representing muscle edema (Figures 1(a), 1(b), and 4(b)). Six lesions in three cases exhibited a small area of long T1 and T2 signal inside the tumor, which represented cystic change and/or necrosis. The two cases with diffusion-weighted imaging showed hyperintensity (Figure 3(d)). The contrast-enhanced scans of four cases demonstrated marked heterogeneous enhancement with band-like areas showing no enhancement, three of which showed a heterogeneous peripheral enhancement (Figure 2(d)) and one a spoke-wheel-like enhancement (Figure 4(c)).

Seven cases demonstrated nodular-like lumps around the deep fascia (Figures 2(b) and 4(a)), while another displayed two lesions growing outward from the deep fascia (Figures 1(a) and 1(b)). One lesion demonstrated slight destruction of the adjacent bone (Figure 3(f)).

Histopathological examinations revealed tumor cells and collagen fibers arranged in crisscrossing bundles, demonstrating a characteristic “herringbone" arrangement (Figure 4(d)).

## 4. Discussion

Until recently, malignant fibrous histiocytomas, malignant schwannomas, or synovial sarcomas were often misdiagnosed as adult fibrosarcomas. However, with the application of modern diagnostic techniques such as immunohistochemistry and electron microscopy, this very rare disease, which accounts for only 1–3.6% of adult sarcomas, can be more accurately diagnosed [2, 3].

Most adult fibrosarcomas are located in the deep muscle or fascia. Tumors often have clear boundaries, with parts of the edges having infiltrative growth. Smaller tumors are often encapsulated, and large tumors are often accompanied by bleeding, necrosis, and cystic change. Histologically, the diagnosis of “fibrosarcoma” was applied only to cases displaying one or more of the following characteristics [7, 8]: (1) hyperchromatic spindled cells depicting no more than moderate pleomorphism, (2) a fascicular “herringbone” pattern, (3) a variable degree of interstitial collagen, (4) no morphologic features of myxofibrosarcoma, low-grade fibromyxoid sarcoma, sclerosing epithelioid fibrosarcoma, or fibrosarcoma arising in dermatofibrosarcoma protuberans, and (5) no expression of any markers other than vimentin or very minimal smooth muscle actin.

Studies have shown that adult fibrosarcomas occur most often in the elderly, with an average affliction age of 50, which is consistent with the findings of the past study [6]. Some studies suggest that the incidence rate between males and females is roughly the same [6]; however, others have suggested it is slightly higher in males [8, 9]. In this study, the ratio of male to female patients was 1 : 4, although with the limited sample size it is not possible to draw any conclusions. The clinical manifestations of an adult soft tissue fibrosarcoma are either painless or painful slow growth of solitary nodules. The patient usually seeks medical treatment after sudden accelerated growth. The cases in this study all involved painless nodules, with the course of disease ranging from three months to six years. The disease can metastasize, with the most common sites being the lungs, liver, and bones; it is only rarely seen in the lymph nodes. The recurrence rate is 12–79% and the five-year survival rate approximately 39–54% [9, 10]. The follow-up data on the patients in this study showed six cases of recurrence (66%) and three cases of lung metastasis (33%).

The etiology of adult fibrosarcoma remains unclear, although some studies have shown that it has a relationship with radiation therapy [6]. Adult fibrosarcoma can also be secondary to skin fibrous sarcomas, solitary fibrous tumors, and differentiated liposarcomas. In this study, there was one case of malignant fibrosarcoma found after the patient underwent surgery for skin dermatosis.

A small number of cases showed no obvious characteristics of this disease in the related MRI imaging, making diagnosis difficult [11]. The authors could find no published reports on adult fibrosarcoma that were conducted using CT and MR imaging data from large numbers of patients. Nevertheless, this study demonstrated the following imaging findings that are suggestive of adult fibrosarcoma.

The lesions showed a low signal on T1WI and mixed signals on T2WI, which were more pronounced on fat saturated T2WI, a finding that is consistent with previous reports [11]. On CT, masses appeared as iso-attenuating soft tissue masses similar to muscle. The majority of cases (8/10) in this study presented with lesions under the deep fascia, with seven being accompanied by nodular lumps around the deep fascia edges, and one case (12.5%) showing the lesion breaking through the deep fascia and invading the subcutaneous tissue. This suggests that the lesions and the deep fascia are closely related. The lesions are invasive, are prone to invading the surface, and can even involve the skin and subcutaneous tissue. Eight cases (80%) showed patch-shaped muscle edema around the tumor, with one involving the adjacent bone, which indicated that it was related to infiltration by the tumor. Some (3/9) moderately to poorly differentiated cases with a small area of cystic change and/or necrosis showed patch-shaped obvious long T1 and T2 signals on MRI and low density on CT. Two cases with diffusion-weighted imaging showed diffusion restriction, which indicated dense cellularity. Soft tissue tumor detected by CT and MRI should first be identified as benign or malignant. All these appearances are highly suggestive of a tumor being malignant.

On nonenhanced CT scans, the masses showed a soft tissue density similar to muscle, with no specific information useful for adult fibrosarcoma identification. However, on MRI, all the cases showed band-like areas of low signal on both T1WI and T2WI, without enhancement on contrast-enhanced MRI. These band-like areas of low signal on the MRI sequences exhibited soft tissue density on CT. Considering both the CT and MRI appearances, it was preoperatively speculated that these band-like areas might be due to a large number of collagen fibers, rather than intratumoral ossification, calcification, hemosiderin, or flow void effects. This speculation was also supported by the pathological findings, which showed tumor cells and collagen fibers arranged in crisscrossing bundles. This feature is highly suggestive of a tumor being fibrous.

Among the four cases with contrast-enhanced scans, three tumor lesions showed heterogeneous peripheral enhancement and one demonstrated a spoke-wheel-like enhancement, consistent with the findings of De Schepper's study, which reported that lesions usually exhibit peripheral enhancement and sometimes a spoke-wheel-like enhancement [11]. The enhancement mechanism is not clear. The authors speculate that the enhancement may be related to the arrangement of collagen fibers within the tumor lesions, while the nonenhancing zone may be related to fibrous septation, cystic change, or necrosis of the lesion.

## 5. Differential Diagnosis

On the basis of previous experience, the appearance of adult fibrosarcomas on CT and MRI images may cause a radiologist to misdiagnose the mass as deep fibromatosis or myxofibrosarcoma. It has been reported that in 86% of fibromatosis cases, T2WI displayed hypointense bands without enhancement [12]. When fibromatosis tumors evolve, collagen deposition increases and cellularity and extracellular spaces decrease, resulting in a decrease in the T2WI signal intensity [13]. Fibromatosis typically exhibits a low signal on T1WI and T2WI, making it easy to diagnose. For poorly differentiated tumors, myxofibrosarcoma should first be ruled out. Myxofibrosarcoma often shows large soft tissue masses in the trunk and limbs of the elderly, with unclear boundaries, bleeding within the tumor, necrosis, extremely uneven signals, and stratification in the necrotic sac [14]. A high proportion of myxofibrosarcoma cases have strong infiltration with invasion into the surrounding tissues; their boundaries are difficult to separate from the invaded tissues [15]. We understand that it is difficult to differentiate these tumors on imaging, although some features, such as lesions with clear boundaries under the deep fascia accompanied by nodular lumps around the deep fascia edges with hypointense bands, shown in all sequences, and the appearance of a spoke-wheel-like enhancement may help to suggest a specific diagnosis.

## 6. Conclusions

In conclusion, although the diagnosis of adult fibrosarcoma depends mainly on pathology, the imaging findings also have value. MRI is the most valuable imaging technique for diagnosing adult fibrosarcoma. The lobulated well-defined lesions displayed hypointense signal on T1WI, heterogeneous low and high intensity signal on T2WI, and diffusion restriction. The band-like areas of low signal on all sequences and the soft tissue density on plain CT indicative of fibrous septation may be a clue to the right diagnosis. Furthermore, the lesions showed heterogeneous peripheral enhancement or spoke-wheel-like enhancement after administration of Gd-DTPA. Such imaging findings are highly suggestive of an adult fibrosarcoma, especially the spoke-wheel-like enhancement feature, which may be helpful for suggesting a specific diagnosis.

In the present study, MR imaging demonstrated the margins and internal structures of the masses more clearly and more precisely than CT imaging. However, CT is very useful for preoperative identification of the band-like areas of low signal on all sequences and representing fibrous septation. This sign is very important for the right diagnosis. Therefore, the combination of CT and MRI examinations is helpful for patients with suspected adult fibrosarcoma.

## Figures and Tables

**Table 1:** Clinical findings in ten patients with adult fibrosarcoma.

Patient/age/sex	Location	Lesion size	Shape/lesion number	Margins	Under deep fascia/nodular-like protrusion/broken through deep fascia	Pathological grade	Follow-up (months)/ recurrence/metastatic sites
		(mm)					
1/47/F	Inner side of the deep fascia of the popliteal fossa	94.8	Lobulated/1	Well-defined	Yes/yes/no	Well-differentiated	20 months/yes/no

2/18/F	Inner side of the deep fascia of the inner muscles of the left calf	51.1	Lobulated/1	Well-defined	Yes/yes/no	Moderately differentiated	30 months/yes/lung

3/43/F	Inner side of the deep fascia of the front outer muscles of the left hip	65.2–104.7	Lobulated/2	Well-defined	Yes/yes/yes	Moderately differentiated	No follow-up

4/45/F	Inner side of the deep fascia of the inner/outer muscles of the right hip	12.1–103.2	Lobulated/9	Well-defined	Yes/yes/no	Moderately differentiated	25 months/yes/no

5/51/F	Inner side of the deep fascia of the back muscles of the left lower thigh	23.8	Oval/1	Well-defined	Yes/yes/no	Moderately differentiated	20 months/yes/lung

6/58/F	Inner side of the deep fascia of the back outer muscles of the left forearm	85.8	Lobulated/1	Well-defined	Yes/yes/no	Moderately differentiated	17 months/no/no

7/46/F	Inner side of the deep fascia of the plantar	43.5	Lobulated/1	Well-defined	Yes/no/no	Poorly differentiated	35 months/no/lung

8/71/F	Muscle gap of the back outer muscles of the left calf	48.7	Lobulated/1	Well-defined	No/no/no	Poorly differentiated	17 months/no/no

9/75/M	Muscle gap of the front inner muscles of the right hip	75.4	Lobulated/1	Well-defined	No/no/no	Moderately differentiated	37 months/yes/no

10/48/F	Inner side of the deep fascia of the upper front muscles of the right thigh	32.1	Lobulated/1	Well-defined	Yes/yes/no	Poorly differentiated	2 months/no/no

**Table 2:** Imaging findings in ten patients with adult fibrosarcoma.

Patient/age/ sex	CT/MRI image	CT Attenuation	MRI					Adjacent bone infiltration	Necrosis or cystic	Fibrous septum
			T1	T2	DWI	Enhancement pattern	Muscles edema			
1/47/F	Yes/yes	Iso	Hypo	Mixed		Peripheral enhancement	No	No	No	Yes

2/18/F	No/yes		Hypo	Mixed		Spokes-like enhancement	Yes	No	No	Yes

3/43/F	No/yes		Hypo	Mixed			Yes	No	No	Yes

4/45/F	No/yes		Hypo	Mixed			Yes	No	Yes	Yes

5/51/F	Yes/yes	Iso	Hypo	Mixed		Peripheral enhancement	Yes	No	No	Yes

6/58/F	Yes/yes	Iso	Hypo	Mixed			Yes	No	Yes	Yes

7/46/F	Yes/yes	Iso	Hypo	Mixed	Hyper		Yes	Yes	No	Yes

8/71/F	No/yes		Hypo	Mixed			Yes	No	Yes	Yes

9/75/M	Yes/no	Iso					----	No	No	Yes

10/48/F	Yes/yes	Iso	Hypo	mixed	Hyper	Peripheral enhancement	Yes	No	No	Yes

Iso: isointense; Hyper: hyperintense; Hypo: hypointense; mixed: mixed hyperintense and hypointense signal intensity; the CT attenuation or MRI intensity is in comparison with adjacent muscle.
